# Hierarchical Sectorized ANN Model for DoA Estimation in Smart Textile Wearable Antenna Array Under Strong Noise Conditions

**DOI:** 10.3390/s25185704

**Published:** 2025-09-12

**Authors:** Zoran Stanković, Olivera Pronić-Rančić, Nebojša Dončov

**Affiliations:** Faculty of Electronic Engineering, University of Niš, A. Medvedeva 4, 18000 Niš, Serbia; olivera.pronic@elfak.ni.ac.rs (O.P.-R.); nebojsa.doncov@elfak.ni.ac.rs (N.D.)

**Keywords:** artificial neural network (ANN), direction of arrival (DoA), textile wearable antenna array (TWAA), SNR, multilayer perceptron (MLP), Root MUSIC

## Abstract

A novel hierarchical sectorized neural network module for a fast direction of arrival (DoA) estimation (HSNN-DoA) of the signal received by a textile wearable antenna array (TWAA) under strong noise conditions is presented. The developed DoA module accounts for variations in antenna element gain, inter-element spacing, and resonant frequencies under the conditions of textile crumpling caused by the motion of the TWAA wearer. The proposed model consists of a sector identification phase, which aims to determine the spatial sector in which the radio gateway (RG) is currently located based on the elements of the spatial correlation matrix of the signal sampled by the TWAA, and a DoA estimation phase, which aims to accurately determine the angular position of the RG in the azimuthal plane. The architecture of the HSNN-DoA module, with different time window lengths in which angular position of RG is recorded, is investigated and compared with the DoA module based on a stand-alone MLP network and the corresponding Root-MUSIC DoA module in terms of accuracy and speed of DoA estimation under variable noise conditions.

## 1. Introduction

Wearable devices support a wide range of applications, including healthcare, entertainment, security, military operations, ambient-assisted living, and sports [1,2,3,4,5]. In healthcare monitoring (HCM), wearable sensors are attached to patients’ bodies, facilitating the continuous collection of critical physiological parameters such as electrocardiogram (ECG) signals, body temperature, heart rate, and respiratory rate. These sensors transmit data to remote monitoring centers via one or more antennas, often integrated into patients’ clothing using various wireless communication technologies [6,7,8,9,10,11]. Furthermore, the incorporation of real-time location systems into HCM solutions enables the tracking of medical staff and patients [12], thereby improving operational visibility and potentially enabling faster response.

Textile-based antennas are particularly well suited for HCM services, because they can be seamlessly integrated into everyday clothing. Textile substrates typically exhibit low dielectric constants and permit the integration of conductive threads—often through manual embroidery. Quasi-omnidirectional textile wearable antennas are often favored for their ability to maintain stable links to radio gateways (RGs) regardless of user posture or movement [13]; however, they typically have low gain [14], which may limit their practical operating range. Multi-element textile wearable antenna arrays (TWAAs) offer a more effective alternative by exploiting the relatively large torso area (e.g., the chest) for antenna placement. TWAAs provide significantly higher gain [11]; however, their narrow and spatially invariant radiation pattern can make them susceptible to signal fluctuations, even when the user is stationary. One solution to maintain continuous alignment between a TWAA and an RG is adaptive beamforming, a functionality that must operate in real-time to be effective. Several well-established algorithms, such as MUSIC and ESPRIT, provide high-accuracy direction of arrival (DoA) estimation [15]. However, these algorithms require computationally expensive matrix operations and, consequently, substantial hardware resources [16,17,18]. This requirement poses a challenge because smart TWAAs must be compact and unobtrusive when integrated into clothing, while minimizing interference with the wearer’s daily activities.

An approach based on Artificial Neural Networks (ANNs) [19,20] has been explored in our previous research for a real-time DoA estimation in smart TWAA [21,22,23,24]. This approach was motivated by two key factors: 1) ANNs can be trained to perform DoA estimation efficiently with minimal hardware resources; and 2) once trained, they achieve accuracy comparable to super-resolution algorithms, while requiring significantly less processing time [21,22,23,24,25,26,27]. In ref. [23], we introduced a basic version of the DoA module based on a MultiLayer Perceptron (MLP) network, referred to as the MLP_DoA module, as an integral component of a smart two-element TWAA DoA subsystem designed for fast DoA estimation. Building upon this, an advanced version of the MLP_DoA module is presented in [24]. That study considers a more realistic scenario where the TWAA comprises two or more antenna elements (multi-element TWAA) and the DoA module accounts for variations in antenna element gain, inter-element spacing, and their resonant frequencies due to textile creasing caused by TWAA wearer motion.

In the presence of strong noise, the accuracy of the DoA module from [24] can be significantly reduced, which may limit its application in real-world scenarios. Therefore, in this paper we present the research results on the further development of an ANN-based DoA module designed to be implemented in an intelligent TWAA DoA subsystem capable of fast and accurate DoA estimation over a wide azimuth range and at low signal-to-noise ratios (SNRs). For this purpose, two different architectures of the DoA module have been developed: one based on a standalone MLP network (MLP-DoA module) and one based on a hierarchical sectorised neural network (HSNN-DoA module), comprising a sector identification phase followed by a DoA estimation phase.

The structure of the paper is as follows: Section 2 introduces the architecture of the proposed smart TWAA DoA subsystem and provides a concise overview of the TWAA signal model. Section 3 focuses on the design of the DoA module, starting with a brief explanation of the basic DoA module based on a standalone MLP network, followed by a detailed description of the newly developed DoA module based on a hierarchical sectorized neural network (HSNN-DoA module). The most illustrative numerical results are presented in Section 4, while concluding remarks are given in Section 5.

## 2. TWAA DoA Subsystem

**Figure 1:**
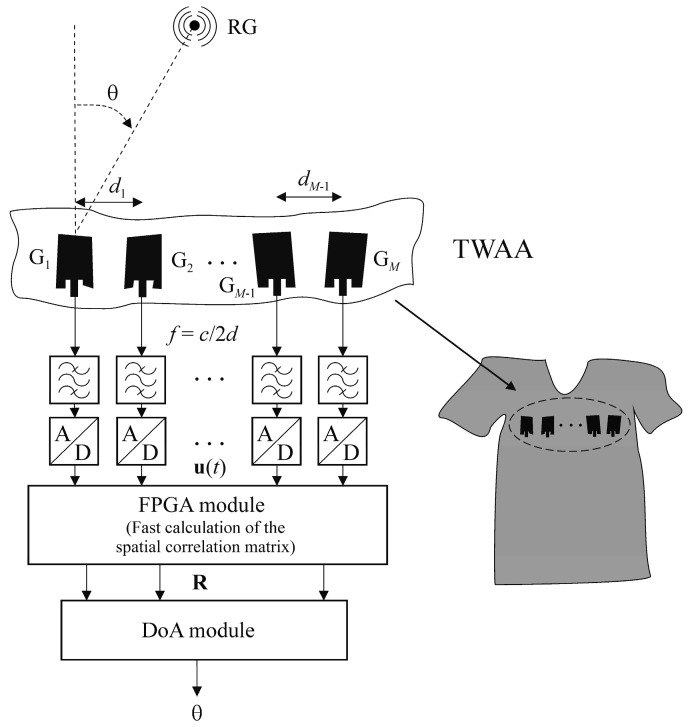
Architecture of the smart TWAA DoA subsystem.

The primary function of the TWAA DoA subsystem is to estimate, at any given time, the angular position of the RG in the azimuthal plane (*θ*) and the TWAA wearer’s current spatial position during motion. The architecture of the smart TWAA DoA subsystem is shown in Figure 1. It comprises a TWAA with *M* elements integrated into the wearer’s upper body clothing, narrow-band filters, analog-to-digital (A/D) converters, a field-programmable gate array (FPGA) module, and a DoA module [24].

The TWAA receives electromagnetic (EM) waves from, and transmits to, the RG at a frequency *f*. When the TWAA wearer is stationary and the textile is not creased, the TWAA behaves as a classic uniform linear antenna array with planar antenna elements of identical geometry and resonant frequency *f*. In this case, the gain of each element in the direction *θ* is the same, *G_i_*(*θ*) = *G_θ_*, *i* = 1, …, *M*. In addition, the distance between neighboring antenna elements is uniform and equal to half the wavelength of the signal, therefore *d_i_ = c/*2*f = d*, *i* = 1, …, *M* − 1, where *d_i_* is the distance between the *i*-th and (*i* + 1)-th antenna elements, while the constant *c* represents the speed of light.

When the TWAA wearer starts to move, the fabric may crease, causing deformations and time-varying changes in the geometry of the TWAA structure. The creasing of the fabric alters the spacing between neighboring antenna elements, and at the observed time *t* this distance is given by

(1) $$d_{i}=k_{i}d=k_{i}\left(t\right)d\;i=1,\ldots ,M-1$$

where *k_i_* = *k_i_*(*t*) is the coefficient of distance shortening between the *i*-th and the (*i* + 1)-th antenna element at time *t*.

The crumpling of the textile also leads to a bending of the antenna array elements, resulting in deviations of the antenna gain in the direction *θ* from the value *G_θ_*. The gain of the individual elements changes independently and randomly over time, expressed as *G_i_* = *G_i_*(*θ*, *t*), *i* = 1, 2, …, *M*. These deformations of the antenna elements also lead to slight variations in their resonant frequencies over time relative to the designed resonant frequency *f*. The time-dependent resonant frequency of the *i*-th element is given by *f_ri_*(*t*) = *f* + Δ*f_ri_*(*θ*), *i* = 1, 2, …, *M*, where Δ*f_ri_*(*θ*) represents the deviation of the resonant frequency of the *i*-th element in the direction *θ*. This slight frequency shift Δ*f_ri_* (according to [28], no more than 5% of the resonant frequency) causes the impedance of the *i*-th antenna element to have a slightly capacitive or inductive character. This in turn leads to a phase shift in the signal received by this antenna element. If two adjacent antenna elements are considered, these phase shifts in the received signals lead to additional variations in the phase difference between the signals received by the neighboring elements.

If *φ****_i_*** denotes the phase difference between the signals received by the *i*-th and (*i* + 1)-th element, at the observed time *t*, it can be expressed as follows:

(2) $$\varphi_{i}(t)=\varphi_{i}^{d}(t)+\varphi_{i}^{f}=\beta \;k_{i}\left(t\right)d\sin\theta +\varphi_{i}^{f}(t),\;i=1,2,\ldots ,M-1$$

where *φ_i_^d^* is the phase difference of the signals received by the *i*-th and (*i* + 1)-th antenna elements caused by the difference in path lengths of the EM waves from the RG to those antenna elements, *φ_i_^f^* is the additional phase variation caused by the fluctuation of the resonant frequency of the antenna elements, and *β* is the phase constant given by *β* = 2π/λ, where λ is the wavelength of the signal.

Based on Equation (2), the effective distances between two adjacent elements of the antenna array at time *t* during the TWAA wearer’s movement can be determined using the following expression:

(3) $$d_{i}^{e}=d_{i}^{e}(t)=\frac{\varphi_{i}(t)}{\beta \sin\theta }=k_{i}\left(t\right)d+\frac{\varphi_{i}^{f}(t)}{\beta \sin\theta }=\;k_{i}^{e}\;\left(t\right)d,\;i=1,2,\ldots ,M-1$$

where *k_i_^e^* = *k_i_^e^*(*t*) = *k_i_*(*t*) + *φ_i_^f^*(*t*)/*βd* is the effective coefficient of the distance shortening between the *i*-th and the (*i* + 1)-th antenna element at time *t*.

The angular position of RG, *θ*, is estimated using the DoA estimation process, which utilizes the information contained in the spatial correlation matrix of the signal, **R**. Under conditions of uncorrelated noise (e.g., white Gaussian noise), **R** is determined by the signal vector at the output of the TWAA, **u**(*t*), as follows [15]:

(4) $$R=E\left[u\left(t\right)u^{H}\left(t\right)\right]=R_{s}+R_{n}=pvv^{H}+\sigma_{n}^{2}I.$$

In Equation (4), *E*[⋅] represents the expectation operator, and **u**(*t*) = **s**(*t*)+ **n**(*t*) denotes the sum of the signal vector **s**(*t*) that would be observed at the output of the TWAA at time *t* in the absence of noise, and the noise vector **n**(*t*) induced on the antenna elements at time *t*. The noise vector can be represented as **n**(*t*) = [*n*_1_(*t*) *n*_2_(*t*) … *n_i_*(*t*) … *n_M_*(*t*)]*^T^*, where *n_i_*(*t*) is the random noise component on the *i*-th antenna element with the variance *σ_n_*^2^. **R***_s_*= *p***vv***^H^* is the spatial correlation matrix of the signal in the absence of noise, and **R***_n_*= *σ_n_*^2^**I** is the spatial correlation matrix of the noise. Here, *p* represents the power of the RG signal induced on an omnidirectional antenna element at the position of the TWAA wearer, while v denotes the steering vector, defined as follows:

(5) $$v={\left[\begin{array}{cccc} \sqrt{G_{1}} & \sqrt{G_{2}}e^{j\beta d_{1}^{e}\sin\theta } & \ldots & \sqrt{G_{M}}e^{j\beta (d_{1}^{e}+d_{2}^{e}+\ldots +d_{M-1}^{e})\sin\theta } \end{array}\right]}^{T},$$

where $\sqrt{G_{i}}$, *i* = 1, 2, …, *M* represents the square root of the gain of the *i*-th antenna element defined in terms of power, often referred to as the amplitude factor of the antenna element. Based on Equation (3), the steering vector can be expressed as follows:

(6) $$v={\left[\begin{array}{cccc} \sqrt{G_{1}} & \sqrt{G_{2}}e^{j\beta d\;k_{1}^{e}\sin\theta } & \ldots & \sqrt{G_{M}}e^{j\beta d(k_{1}^{e}+k_{2}^{e}+\ldots +k_{M-1}^{e})\sin\theta } \end{array}\right]}^{T}.$$

Using expressions (4) and (6), as well as defining the *SNR* in relation to the power of the signal received by the first element of the antenna array, *SNR* = (*G*_1_*p*)/*σ_n_*^2^, the spatial correlation matrix **R** can be expressed as follows:

(7) $$R=\left[\begin{array}{cccc} G_{1}p+\frac{G_{1}p}{SNR} & \sqrt{G_{1}G_{2}}pe^{-j\beta d\cdot k_{1}^{e}\sin\theta } & \ldots & \sqrt{G_{1}G_{M}}pe^{-j\beta d\left(k_{1}^{e}+\ldots +k_{M-1}^{e}\right)\;\sin\theta }\\ \sqrt{G_{1}G_{2}}pe^{j\beta d\cdot k_{1}^{e}\sin\theta } & G_{2}p+\frac{G_{1}p}{SNR} & \ldots & \sqrt{G_{2}G_{M}}pe^{-j\beta d\left(k_{2}^{e}+\ldots +k_{M-1}^{e}\right)\;\sin\theta }\\ \vdots & \vdots & \ddots & \vdots \\ \sqrt{G_{1}G_{M}}pe^{j\beta d\left(k_{1}^{e}+\ldots +k_{M-1}^{e}\right)\;\sin\theta } & \sqrt{G_{2}G_{M}}pe^{j\beta d\left(k_{2}^{e}+\ldots +k_{M-1}^{e}\right)\;\sin\theta } & \ldots & G_{M}p+\frac{G_{1}p}{SNR} \end{array}\right].$$

When the spatial correlation matrix **R** is normalized with respect to the element *R*_11_, a normalized correlation matrix **R**′ is obtained, which leads to the same results in DoA estimation as the non-normalized matrix **R** [21,23]. Normalization offers two main advantages: First, it eliminates the need to know the signal power *p*. Second, it eliminates the need to know the gains of all antenna elements, since it is sufficient to know the gain ratio of the *i*-th (*i* = 2, 3, …, *M*) and the first antenna element, *G_i_*/*G*_1_. If we introduce the variable *g**_i_***—the root gain ratio of the *i*-th antenna element—as $g_{i}=\sqrt{G_{i}/G_{1}}$ and express the distance between the antenna elements on non-creased textile, *d*, in terms of wavelengths, *d_λ_*, the normalized spatial correlation matrix can be expressed as follows:

(8) $$R^{\prime }=\left[\begin{array}{cccc} 1 & g_{2}\cdot \frac{SNR}{SNR+1}pe^{-j2\pi d_{\lambda }\cdot k_{1}^{e}\;\sin\theta } & \ldots & g_{M}\cdot \frac{SNR}{SNR+1}pe^{-j2\pi d_{\lambda }\left(k_{1}^{e}+\ldots +k_{M-1}^{e}\right)\;\sin\theta }\\ g_{2}\cdot \frac{SNR}{SNR+1}pe^{j2\pi d_{\lambda }\cdot k_{1}^{e}\sin\theta } & \frac{SNR}{SNR+1}\left(g_{2}^{2}+\frac{1}{SNR}\right) & \ldots & g_{2}\cdot g_{M}\cdot \frac{SNR}{SNR+1}pe^{-j2\pi d_{\lambda }\left(k_{2}^{e}+\ldots +k_{M-1}^{e}\right)\;\sin\theta }\\ \vdots & \vdots & \ddots & \vdots \\ g_{M}\cdot \frac{SNR}{SNR+1}pe^{j2\pi d_{\lambda }\left(k_{1}^{e}+\ldots +k_{M-1}^{e}\right)\;\;\sin\theta } & g_{2}\cdot g_{M}\cdot pe^{j2\pi d_{\lambda }\left(k_{2}^{e}+\ldots +k_{M-1}^{e}\right)\;\sin\theta } & \ldots & \frac{SNR}{SNR+1}\left(g_{M}^{2}+\frac{1}{SNR}\right) \end{array}\right]$$

The DoA module for determining the angle *θ* uses the values of the elements in the matrix **R**′. However, in the scenario of the movement of the TWAA wearer, the determination of the matrix **R**′ by directly applying the Equation (8) is not possible for two reasons. The first reason is that the textile is randomly crumpled during the movement of the TWAA wearer, which leads to a random time variation of the parameters *k_i_^e^* and *g_i_*. The second reason is that the angle *θ* is unknown (DoA estimation is an inverse problem compared to determining the spatial correlation matrix based on the angle *θ*).

For the reasons mentioned above, the correlation matrix at the observed time moment *t* is estimated from a large number of TWAA output samples within a short time interval [*t*, *t* + Δ*t*] (TWAA snapshots), using fast A/D converters. The matrix elements are then calculated on the FPGA module using the following approximate formula [23]:

(9) $$R(t)\approx \frac{1}{N_{ss}}\sum_{ss=1}^{N_{ss}}u_{ss}u_{ss}^{H}\;\left(normalized\;matrix:\;R^{\prime }(t)=\frac{1}{R_{11}}R(t)\right)$$

where *N_ss_* is the number of snapshots and **u***_ss_* is the sample of the *ss*-th snapshot of the **u**(*t*) signal, which represents the TWAA output.

The estimation of the spatial correlation matrix **R** at the observed time *t* based on Equation (9) represents sampling of the values of this matrix, and the value **R**(*t*) represents the matrix sample at time *t*.

To simulate the movement of the TWAA wearer in the RG environment, signal sampling at the TWAA elements, and the estimation of spatial correlation matrix elements in the presence of noise based on TWAA snapshots, the *TWAA_Sim_Cmtx* simulation software was developed in the MATLAB (R2020a) environment. The core of this software is based on the TWAA signal model presented in this section.

## 3. DoA Module

During the movement of a TWAA wearer along a trajectory, the relative angular position of RG in the azimuthal plane with respect to the antenna array changes with time, *θ* =*θ* (*t*). The task of the DoA module is to determine the angle *θ* at a given time *t* based on the values of the elements of the normalized spatial correlation matrix **R**′ estimated at the observed time *t*; that is, to perform the following mapping:

(10) $$\theta (t)=f_{DoA}(R^{\prime }(t)).$$

### 3.1. DoA Module Based on Standalone MLP Network (MLP-DoA Module)

**Figure 2:**
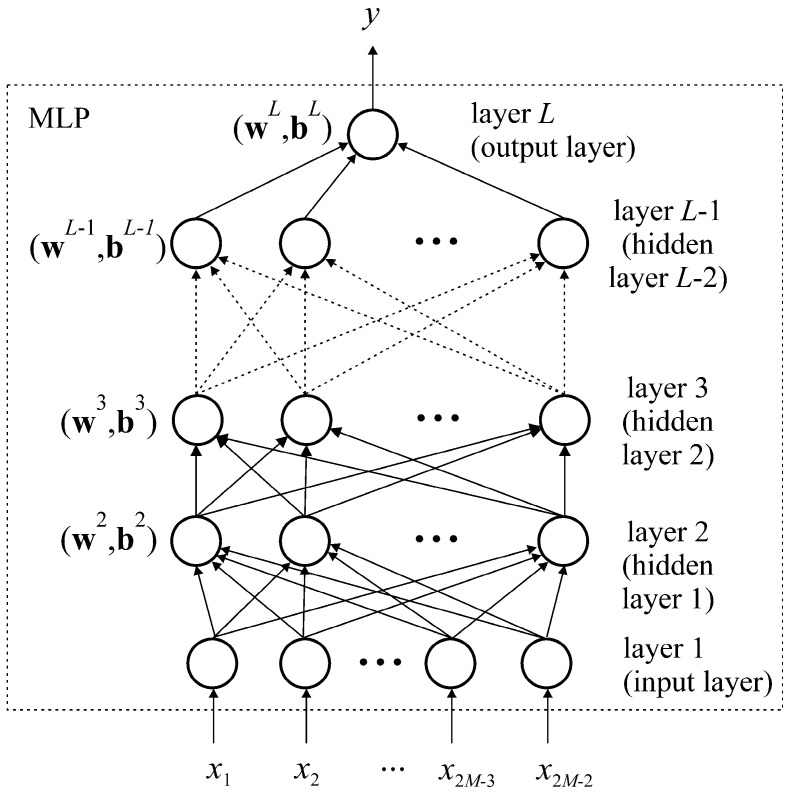
Architecture of the MLP network.

The MLP-DoA module is implemented as a standalone MLP network whose architecture is shown in Figure 2. In the previous papers [21,23], it has been demonstrated that the values of the elements in the first row of matrix **R**′, excluding the autocorrelation element, are sufficient for the accurate estimation of angular positions of EM radiation sources when using MLP networks. However, due to the limitation that MLP networks do not support complex-valued inputs, the input variable vector for the MLP network takes the following form:

(11) x=[x1x2…x2M−2]=ReR′12ImR′12ReR′13ImR′13…ReR′1MImR′1M

The presented network architecture consists of a total of *L* layers of neurons, including one input layer, *L* − 2 hidden layers, and one output layer. The input layer serves as a buffer layer and contains 2*M* − 2 neurons. Consequently, its output vector is identical to the input variable vector, i.e., **y**^1^ = **x**.

The output vectors of all other layers in the network can be expressed as follows:

(12) $$y^{l}=F_{l}(w^{l}y^{l-1}+b^{l})\;l=2\;\;,3,\ldots ,L$$

where ***F****_l_*(⋅) is the activation function of the neurons in the *l*-th layer, **w***^l^* is the connection weight matrix between the (*l* − 1)-th and the *l*-th layer (the matrix element *w^l^_i_*_,*j*_ represents the connection weight between the *j*-th neuron of the (*l* − 1)-th layer and the *i*-th neuron of the *l*-th layer), and **b***^l^* is the bias vector of the *l*-th layer (the vector element *b_i_^l^* represents the bias of the *i*-th neuron of the *l*-th layer). The hyperbolic tangent sigmoid transfer function is chosen as the activation function for the hidden layers: (F*_i_*(*u*) = (*e^u^*− *e^−u^*)/(*e^u^* + *e^−u^*), *i* = 2, 3, …, *L* − 1). For the output layer, a linear activation function is used: (*F_L_*(*u*) = *u*). The output layer consists of a single neuron that provides the angle *θ* (*y* = *θ*). Therefore, the output of the MLP neural network is computed as follows:

(13) $$\theta =y^{L}=F_{L}(w^{L}y^{L-1}+b^{L})=w^{L}y^{L-1}+b^{L}$$

The set of adjustable parameters of an MLP neural network, *W*, comprises the weight matrices **w**^2^, **w**^3^, …, **w***^L^*, and the bias vectors **b**^2^, **b**^3^, …, **b***^L^*. During the network training, these parameters are iteratively updated to ensure that the network achieves the mapping described by Equation (10) with the required precision.

The training of the MLP network in the MLP-DoA module (MLP_DoA network) is performed by Levenberg–Marquardt (LM) algorithm [20] using training, test, and validation datasets. The training dataset has the format *P* = *P*(*SNR*, *M*) = {(**x**_1_,*θ*_1_*^D^*), (**x**_2_,*θ*_2_*^D^*), …, (**x***_p_*,*θ_p_^D^*), …, (**x***_Np_*,*θ_Np_^D^*)}, where *θ_p_^D^* is the desired value at the network output when the input vector of the *p*-th sample (**x***_p_*) is present at the network input, and *N_p_* is the number of training samples. The input vector of the network for the *p*-th training sample is formatted as follows:

(14) xp=ReR′12(tp,SNR)ImR′12(tp,SNR)…ReR′1M(tp,SNR)ImR′1M(tp,SNR)

where *R*′_12_(*t_p_*, *SNR*),…, *R*′_1*M*_(*t_p_*, *SNR*) are the complex values of the elements in the first row of the correlation matrix **R**′(*t*), estimated at time *t* = *t_p_* during the movement of the TWAA wearer under noise conditions defined by the signal to noise ratio, *SNR*, as follows:

(15) $$R^{\prime }(t_{p},SNR)=R^{\prime }(\theta_{p}^{D}(t_{p}),\;d_{\lambda },\;k_{1}^{e}(t_{p}),\ldots ,k_{M-1}^{e}(t_{p}),\;g_{2}(t_{p}),\ldots ,g_{M}(t_{p}),\;SNR).$$

The test set *T* has the same format as the training set *P*, with the exception that the total number of samples in the test set is *N_T_*. For the validation set, *V*, the same set as the test set is used during the training of the MLP network, i.e., *V* = *T*, *N_V_* = *N_T_*.

The TWAA_Sim_Cmtx simulation software was developed to create samples for the training and the test sets. This program assumes that the textile deforms randomly during the movement of the TWAA wearer. As a result, at the observed sampling time *t_p_*, the parameter values *k*_1_*^e^*(*t_p_*), *k*_2_*^e^*(*t_p_*), …, *k_M_*_−1_*^e^*(*t_p_*) and *g*_2_(*t_p_*), *g*_3_(*t_p_*), …, *g_M_*(*t_p_*) have random values with a uniform distribution within the ranges [*k^e^*_min_, *k^e^*_max_] and [*g*_min_, *g*_max_], whereby the range limits can be defined in the program. In addition, when generating samples, the sampling time *t_p_* is also selected randomly, which means that the values of *θ_p_^D^* follow a uniform distribution within the range [*θ*_min_, *θ*_max_].

During the training of the MLP network, the level of its generalization capabilities is observed, which is represented by the mean squared error of the network output *θ* on the validation set for the current value of the trainable parameters *W*. Training continues as long as this error decreases. When the error reaches its minimum value, defined as

(16) $$E_{V\min}\left(W\right)=\underset{W}{\min}\left(\frac{1}{2N_{V}}\sum_{s=1}^{N_{V}}{\left(\theta_{p}-\theta_{p}^{D}\right)}^{2}\right),$$

it is assumed that the MLP network has reached its maximum generalization capabilities, and the training is terminated. In practice, to confirm that a global minimum of the error in the validation set has been reached and that it is not just a local oscillation of the error, training continues for further MVF (maximum validation failure) iterations after reaching the minimum, where the error must remain higher than the minimum.

After the completion of training, the MLP network is tested to assess the quality of its training. The following metrics were used to evaluate the quality of the network’s training on the test set: worst-case error (*WCE*), average test error (*ATE*), Pearson product-moment correlation coefficient (*r^PPM^*), and root mean square error (RMSE) [19].

The worst-case error is calculated by the following formula:

(17) $$WCE=\overset{N_{T}}{\underset{p=1}{\max}}\left|\frac{\theta \left(x_{p},W\right)-\theta_{p}^{D}}{\theta_{\max}^{D}-\theta_{\min}^{D}}\right|$$

where *N_T_* is the total number of samples in the test set, *θ* (**x***_p_*, *W*) is the output of the MLP_DoA network for the input sample **x***_p_*, *θ_p_^D^* is the desired value of the network output for the sample **x***_p_*, and *θ ^D^*_max_ and *θ ^D^*_min_ represent the maximum and minimum desired values of the angle *θ* in the test set, respectively.

The average test error is calculated as follows:

(18) $$ATE=\frac{1}{N_{T}}\sum_{p=1}^{N_{T}}\left|\frac{\theta \left(x_{p},W\right)-\theta_{p}^{D}}{\theta_{\max}^{D}-\theta_{\min}^{D}}\right|.$$

Pearson product-moment correlation coefficient is calculated as follows:

(19) $$r^{PPM}=\frac{\sum_{p=1}^{N_{T}}\left(\theta \left(x_{p},W\right)-\bar{\theta }\right)\cdot \left(\theta_{p}^{D}-{\bar{\theta }}^{D}\right)}{\sqrt{\left[\sum_{p=1}^{N_{T}}{\left(\theta \left(x_{p},W\right)-\bar{\theta }\right)}^{2}\right]\cdot \left[\sum_{p=1}^{N_{T}}{\left(\theta_{p}^{D}-{\bar{\theta }}^{D}\right)}^{2}\right]}},$$

where $\bar{\theta }=\frac{1}{N_{T}}\sum_{p=1}^{N_{T}}\theta \left(x_{p},W\right)$ is the average value of neural network output and ${\bar{\theta }}^{D}=\frac{1}{N_{T}}\sum_{p=1}^{N_{T}}\theta_{p}^{D}$ is the average value of the desired output values.

The *RMSE* value of the output of the neural network on the test set is defined as

(20) $$RMSE=\sqrt{\frac{1}{N_{T}}\sum_{p=1}^{N_{T}}{\left(\theta \left(x_{p},W\right)-\theta_{p}^{D}\right)}^{2}}.$$

### 3.2. DoA Module Based on Hierarchical Sectorized Neural Network (HSNN-DoA Module)

The HSNN-DoA module is based on the subdivision of the angular azimuth space into *N_S_* equal, non-overlapping angular sectors [*θ_s_*_min_, *θ_s_*_max_], where *s* ∈ {1,2, …, *N_S_*}. The boundaries of the *s*-th sector, *θ_s_*_min_ and *θ_s_*_max_, are defined as *θ_s_*_min_ = *θ*_min_ + (*s* − 1)Δ*θ* and *θ_s_*_max_ = *θ_s_*_min_ + Δ*θ*, where *θ*_min_ and *θ*_max_ are the minimum and maximum values of the angular position of RG relative to TWAA, respectively, and Δ*θ* is the angular width of the sector defined as Δ*θ* = (*θ*_max_ − *θ*_min_)/*N_S_*. At the beginning, the relative angular position of RG to TWAA at time *t* is determined, or simply which sector it belongs to at time *t*. In the second phase, after determining that RG is in the *s*-th sector, the determination of the angle *θ*(*t*) is performed by a specialized MLP_DoA network associated with the *s*-th sector (a network trained for DoA estimation only for the *s*-th sector). If it is not possible to determine in which sector RG is currently located due to strong noise or inaccuracies of the neural model, the current angular position of RG is assumed to be the position observed at the previous moment (*θ*(*t*) = *θ*(*t* − 1)).

**Figure 3:**
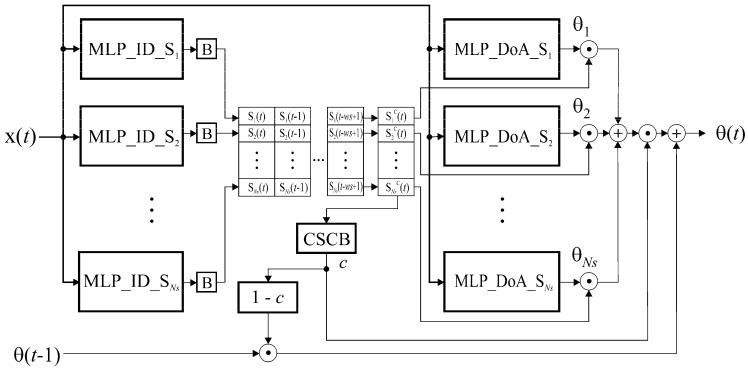
Architecture of the HSNN-DoA module.

In accordance with the above-mentioned concept, the HSNN-DoA module consists of two phases, namely the sector identification phase and the DoA estimation phase. The architecture of this module is shown in Figure 3.

The sector identification phase determines the spatial sector in which the RG is currently located. It comprises a total of *N_S_* MLP networks with the designation MLP_ID_S*_i_*, *i* = 1, 2, …, *N_S_* (the *i*-th network is assigned to the *i*-th sector); a total of *N_S_* functional blocks (B) for creating a binary decision, whereby each MLP_ID_S*_i_* network is assigned to one B block; and memory space for storing the history of the binary decisions of the MLP_ID_S*_i_* networks in *ws* consecutive time points (output vectors of the networks for the current time point *t* and *ws* − 1 previous time points).

Each MLP_ID_S*_i_* network has the task of determining whether the RG is in its sector or not. It is trained to generate an output value greater than or equal to 0.5 for a given input sample **x**(*t*) if the RG is in its sector, or a value less than 0.5 if the RG is not in its sector. As the output of each MLP network is analog and therefore not suitable for further binary processing, it is converted into a binary decision using a B block. The transfer function of the B-block is described by the following expression:

(21) $$S_{i}=f_{Bi}(f_{MLP\_ID\_Si}(x(t)))=\left\{\begin{array}{c} 1,\;f_{MLP\_ID\_Si}(x(t))\geq 0.5\\ 0,\;f_{MLP\_ID\_Si}(x(t))<0.5 \end{array}\right.\;,$$

where *S_i_* represents the output of the B-block associated with sector *i* and *f_MLP_ID_Si_* (·) is the transfer function of the MLP_ID_S*_i_* network. For the sample **x**(*t*), which is fed to the inputs of all MLP_ID_S*_i_* networks, the values *S_i_*, *i* = 1, 2, …, *N_S_*, form the binary output vector of the networks, **S**(*t*).

Under increased noise conditions, the probability that one of the MLP_ID_S*_i_* neural networks makes a wrong decision (i.e., to decide that the RG is in its sector even though it is in another sector, or to decide that the RG is not in its sector even though the RG is present in its sector) also increases. In order to reduce the influence of such errors on the accuracy of the sector identification procedure, a strategy was chosen according to which the MLP_ID_S*_i_* networks must confirm the presence of RG in a sector for a series of *ws* time-consecutive samples (**x**(*t* − *ws* + 1), **x**(*t* – *ws* + 2), …, **x**(*t* − 1), **x**(*t*)) that are fed to the input of the DoA module. By following the history of decisions of MLP_ID_S*_i_* neural networks in a time window of length *ws* and making an intersection of those decisions over time, the cumulative sector vector, **S***^c^*, is formed as follows:

(22) $$S^{c}(t)=S(t)\circ S(t-1)\circ \ldots \circ S(t-ws+1)$$

where the operator “ο“ represents element-wise vector multiplication. 

If this binary vector has only one element that is equal to “1”, then the decisions of the neural networks agree with each other, i.e., they have made the decision by consensus. This decision is “RG is located in the *s*-th sector, where *s* is the index of the vector **S***^c^* (the position within the vector **S***^c^*) in which the value “1” is obtained.” If the vector **S***^c^* does not have a single element that is equal to “1” (the values of all elements of the vector are “0”), or if the vector has two or more elements whose values are equal to “1”, then the neural networks are not in a consensus state, i.e., they could not agree on the decision as to which sector the RG is in. It is then assumed that the neural networks have not made any decision. These situations occur when the network makes the wrong decision due to increased noise and when the RG is in the border area between two sectors.

The decision on whether the MLP_ID_S*_i_* networks have reached a consensus or not is made by a functional block called the Consensus State Check Block (CSCB). The value “1” appears at the output of this block, *c*, if the neural networks have reached the consensus. If, on the other hand, the value “0” appears at the output of this block, this means that the neural networks are not in consensus and therefore no decision has been made. The processing function of this block is given by

(23) $$c=f_{CSCB}(S^{c}(t))=\left\{\begin{array}{c} 1\;,\;nnz\;(S^{c}(t))=1\\ 0\;,\;nnz\;(S^{c}(t)))\neq 1 \end{array}\right.$$

where the function nnz(**S***^c^*(*t*)) determines the number of non-zero elements in the binary vector **S***^c^*(*t*).

The DoA estimation phase consists of a total of *N_S_* MLP networks, denoted as MLP_DoA_ S*_i_*, *i* = 1, 2, …, *N_S_*, where the *i*-th network is assigned to the *i*-th sector. When a sample **x**(*t*) is provided at the input of the DoA module and the sector identification stage determines that the RG is in the *i*-th sector, the *i*-th MLP_DoA network is activated. This network has the task of estimating the angular position of the RG relative to TWAA. If there is no consensus among the MLP_ID_S*_i_* networks on which sector the RG is in (*c* = 0), the angle determined at the previously observed moment is taken as its current position (*θ*(*t*) = *θ*(*t* − 1)).

The outputs of all MLP_DoA_ S*_i_* networks form a vector **Θ** = **Θ**(*t*) = **Θ**(**x**(*t*)) when the sample **x**(*t*) is entered into the HSNN-DoA module, whereby the value of the *i*-th element of this vector contains the output of the MLP_DoA_ S*_i_* network. Based on the values of the vector **S***^c^* and the output of the CSCB block (value *c*), the final output of the HSNN-DoA module is determined as follows:

(24) $$\theta (t)=(1-c)\cdot \theta (t-1)+c\cdot [S^{c}(t)\Theta (t)]$$

The training and testing procedures for the MLP_ID_S*_i_* and MLP_DoA_ S*_i_* networks follow an approach like that of a standalone MLP network in the MLP_DoA module (Section 3.1), with the following differences: First, the format of the training and testing samples for the MLP_ID_S*_i_* network is (**x***_p_*, *y_p_^D^*), where the desired output value *y_p_^D^* is “1” if the sample **x***_p_* was generated while the RG was in the sector associated with the MLP_ID_S*_i_* network, and *y_p_^D^* is “0” if the sample was generated when the RG was outside this sector. Further, during testing, each MLP_ID_S*_i_* network operates in conjunction with its corresponding B block, using accuracy as the testing metric. Accuracy is calculated as follows: (Number of correct sector classifications/Total test samples) × 100%. Finally, the MLP_DoA_ S*_i_* network is trained and tested exclusively on samples from its associated sector.

## 4. Modeling Results

The numerical results presented here refer to DoA estimation in a smart TWAA system with *M* = 4 antenna elements. All mentioned DoA modules are designed to operate in [−80°, 80°] angular azimuth space. The first part of the section (Section 4.1) describes the development of the MLP-DoA module using an MLP network with two hidden layers. The second part (Section 4.2) presents the development of the HSNN-DoA module using multiple MLP networks with two hidden layers. In the construction of this module, the case that the angular azimuth space [−80°, 80°] is divided into four equal angular sectors (*N_S_* = 4) was considered: [−80°, −40°] (Sector 1), [−40°, 0°] (Sector 2), [0°, 40°] (Sector 3), and [40°, 80°] (Sector 4). In the last part of the section (Section 4.3), the accuracy and speed of DoA estimation using the MLP-DoA module, the HSNN-DoA module, and the Root-MUSIC module are compared while the TWAA wearer moves along the reference trajectory under different noise conditions (different *SNR* values).

The training and testing of the MLP networks during the creation of the DoA modules was carried out with samples generated under low-noise conditions (*SNR* = 20 dB). The notation used for the two-layer MLP network architecture is MLP2-*H*_1_-*H*_2_, where *H*_1_ stands for the number of neurons in the first hidden layer and *H*_2_ for the number of neurons in the second hidden layer.

### 4.1. MLP-DoA Module Development

To identify the optimal standalone MLP network architecture for implementing the MLP-DoA module (MLP-DoA network) with minimum DoA estimation error, a large number of MLP2-*H*_1_-*H*_2_ networks were trained and tested, where *H*_1_, *H*_2_ ∈ [8, 24]. To construct the training set *P*^(20dB)^ = *P*(20 dB, 4) and the test set *T*^(20dB)^ = *T*(20 dB, 4), independent training and test datasets, *P_i_*^(20dB)^ and *T_i_*^(20dB)^, *i* = 1, 2, 3, 4, were first generated for the four above-mentioned angular sectors using the following parameter ranges: [*k^e^*_min_, *k^e^*_max_] = [0.3, 0.5], [*g*_min_, *g*_max_] = [−10 dB, 10 dB], [*θ*_1min_, *θ*_1max_] = [−80°, −40°], [*θ*_2min_, *θ*_2max_] = [−40°, 0°], [*θ*_3min_, *θ*_3max_] = [0°, 40°], and [*θ*_4min_, *θ*_4max_] = [40°, 80°]. Each training and test dataset for a single sector contained 20,000 and 3000 samples, respectively. The full training set for MLP network training was formed as the union of the sector-based training datasets, $P^{\left(20dB\right)}=\overset{4}{\underset{i=1}{\cup }}P_{i}^{\left(20dB\right)}$, while the test set was formed in a similar manner as $T^{\left(20dB\right)}=\overset{4}{\underset{i=1}{\cup }}T_{i}^{\left(20dB\right)}$. The training dataset thus contained 80,000 samples, while the test dataset contained 12,000 samples. Training of the MLP-DoA networks was performed using the LM algorithm with an initial combination coefficient μ^(0)^ = 0.001, while the increase and decrease factors were μ_inc_ = 10 and μ_dec_ = 0.1, respectively. The following training parameters were chosen: *N_Imax_* = 1000 and *MVF* = 20. Testing results for six MLP_DoA networks with the best test statistics are presented in Table 1. The MLP2-23-23 network was selected for implementing the MLP-DoA module, as it demonstrated the lowest *ATE* value.

### 4.2. HSNN-DoA Module Development

In the first step of the HSNN-DoA module development, the sector identification phase was formed by selecting MLP_ID_S*_i_*, *i* = 1, 2, 3, 4 networks. For each sector, multiple MLP2-*H*_1_-*H*_2_ networks (*H*_1_, *H*_2_ ∈ [8, 24]) were trained and tested using the LM training algorithm under the same conditions as for the MLP networks in Section 4.1. During the training and testing of the MLP networks for the *i*-th sector, modified training and test sets, *P_m(i)_*^(20dB)^ and *T_m(i)_*^(20dB)^, containing 80,000 and 12,000 samples, respectively, were used. These modified sets were derived from the original *P*^(20dB)^ and *T*^(20dB)^ sets by replacing the desired value *θ_p_^D^* of the *p*-th sample (*p* = 1, 2, …, *N_P_* for the training set and *p* = 1, 2, …, *N*_T_ for the test set) by “1” if *θ_p_^D^* belongs to the *i*-th sector, or by “0” if not. The output of each MLP_ID network is processed by the functional block B to obtain the final binary output value, which was then used during the network testing.

Table 2 shows the test results for the six MLP networks with the best performance for each individual sector. The MLP2-18-16, MLP2-22-22, MLP2-10-6, and MLP2-9-5 networks were selected for MLP_ID_S_1_, MLP_ID_S_2_, MLP_ID_S_3_, and MLP_ID_S_4_, respectively, for the sector identification phase implementation, as they had the highest accuracy values within their respective groups.

In the second step of the HSNN-DoA module development, the DoA estimation phase was formed by selecting MLP_DoA_S*_i_*, *i* = 1, 2, 3, 4 networks. Like the first step, multiple MLP2-*H*_1_-*H*_2_ networks (*H*_1_, *H*_2_ ∈ [8, 24]) were trained and tested for each sector using the LM training algorithm and under the same conditions as the MLP networks in Section 4.1. During the training and testing of the MLP networks for the *i*-th sector, the training and test sets *P_i_*^(20dB)^ and *T_i_*^(20dB)^ were used, containing 20,000 and 3000 samples, respectively.

Table 3, Table 4, Table 5 and Table 6 contain the test results of the six MLP networks with the best performance for Sectors 1, 2, 3, and 4, respectively. The MLP2-12-8, MLP2-10-6, MLP2-9-7, and MLP2-12-11 networks were selected for MLP_DoA_S_1_, MLP_DoA_S_2_, MLP_DoA_S_3_, and MLP_DoA_S_4_ respectively, for the implementation of the DoA estimation phase, as they had the highest accuracy values within their respective groups. The network with the smallest ATE value was selected as having the highest accuracy.

### 4.3. DoA Module Comparison

To compare the accuracy and speed of DoA estimation with the MLP-DoA module, the HSNN-DoA module, and the Root-MUSIC DoA module, a simulation of the movement of the TWAA wearer along a selected reference trajectory was performed. During this motion, the angular position of the RG relative to the TWAA, *θ*, was determined with each of the DoA modules at 8001 time-equidistant points along the trajectory. The simulation of the motion and the estimation of the angle *θ* with the DoA modules was performed for different *SNR* values along the reference trajectory, namely for *SNR* ∈ [20 dB, 15 dB, 10 dB, 5 dB, 0 dB, −5 dB, −10 dB, −15 dB]. The selected reference trajectory is described by the following formula: *θ =* 10·(*t* − 4)^2^ − 80, *t* ∈ [0, 8], where *t* is the time expressed in time units (TU). When selecting the HSNN-DoA module, three different cases were considered regarding the time integration window length: *ws* = 1, *ws* = 2 and *ws* = 3.

**Figure 4:**
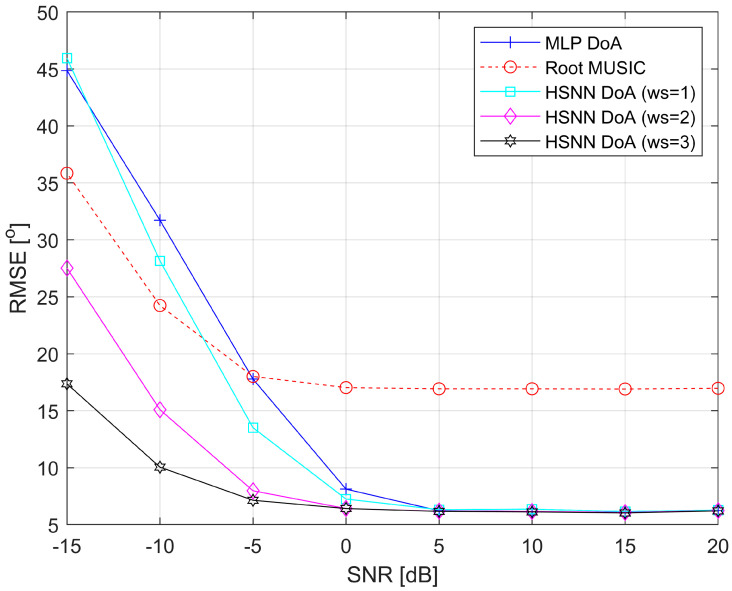
RMSE versus *SNR* obtained by using MLP-DoA module, HSNN-DoA module, and Root-MUSIC DoA module.

Figure 4 illustrates the dependence of the RMSE values on the *SNR* values obtained with the MLP-DoA, HSNN-DoA (*ws* = 1), HSNN-DoA (*ws* = 2), HSNN-DoA (*ws* = 3), and Root-MUSIC DoA modules when estimating the angle *θ* along the reference trajectory. The analysis of the results shows that the MLP-DoA and HSNN-DoA modules in the *SNR* range [5 dB, 20 dB] have approximately the same accuracy in DoA estimation, which is significantly higher than that of the Root-MUSIC DoA module. In the increased noise range (*SNR* ∈ [−5 dB, 5 dB]), a deterioration in the accuracy of the MLP-DoA and HSNN-DoA modules can be observed; however, their accuracy remains higher than that of the Root-MUSIC DoA module. The deterioration in accuracy is more emphasized for the MLP-DoA and HSNN-DoA (*ws* = 1) modules than for the HSNN-DoA (*ws* = 2) and HSNN-DoA (*ws* = 3) modules. The Root-MUSIC DoA module retains approximately the same accuracy as under low-noise conditions. At very high noise levels (*SNR* ∈ [−15 dB, −5 dB]), significant accuracy degradation can be observed for all DoA modules. However, the trend of accuracy deterioration with decreasing *SNR* is much more moderate for the HSNN-DoA (*ws* = 2) and HSNN-DoA (*ws* = 3) modules than for the MLP-DoA, HSNN-DoA (*ws* = 1), and Root-MUSIC DoA modules. Overall, the degradation in DoA estimation accuracy with decreasing *SNR* is least emphasized for the HSNN-DoA (*ws* = 3) module, making it the most resilient to increased noise power.

**Figure 5:**
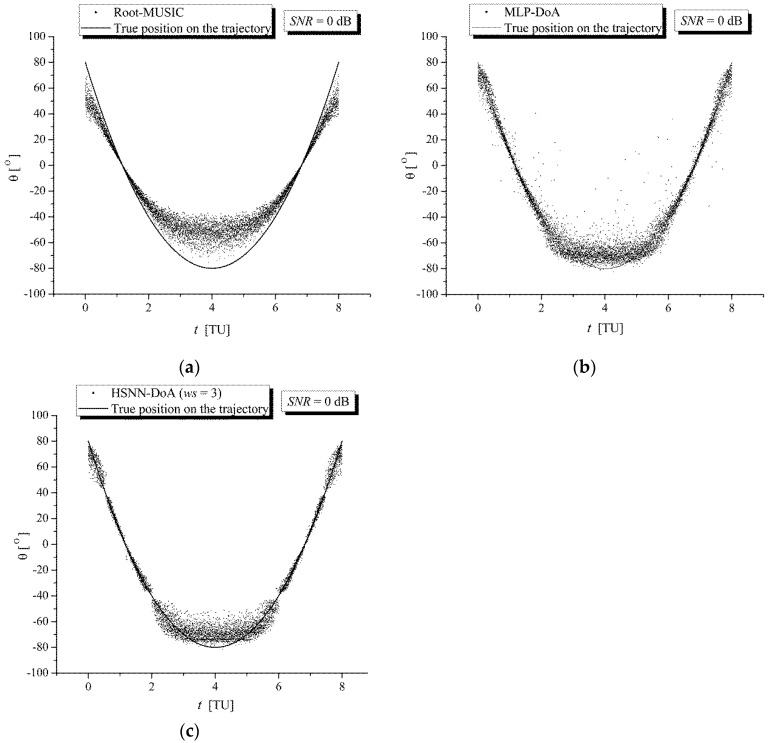
Comparison of DoA estimation results for RG signals along the trajectory of the TWAA wearer obtained with (**a**) the Root-MUSIC module, (**b**) the MLP-DoA module, and (**c**) the HSNN-DoA (*ws* = 3) module for *SNR* = 0 dB.

**Figure 6:**
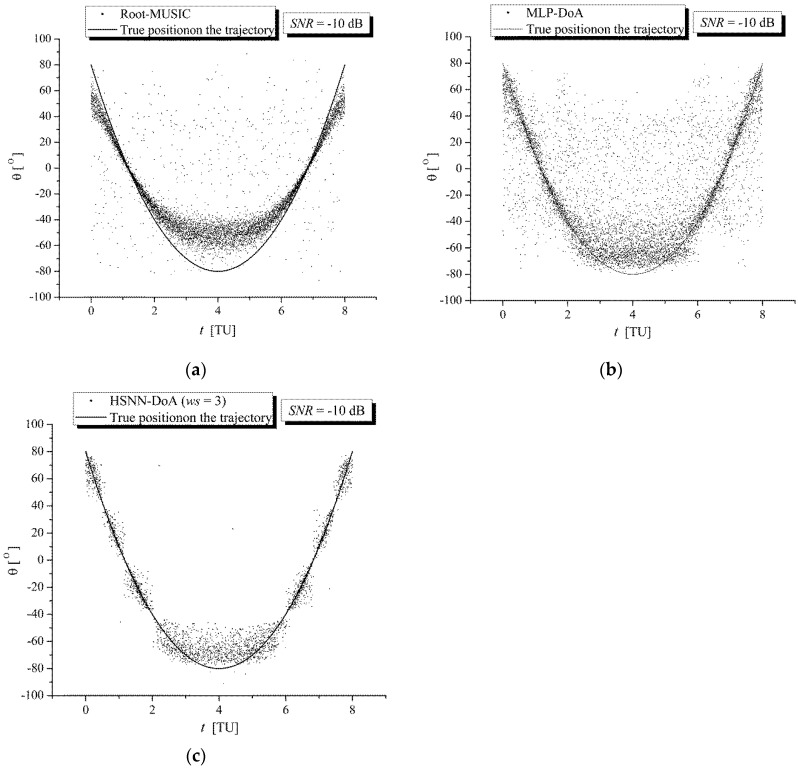
Comparison of DoA estimation results for RG signals along the trajectory of the TWAA wearer obtained with (**a**) the Root-MUSIC module, (**b**) the MLP-DoA module, and (**c**) the HSNN-DoA (*ws* = 3) module for *SNR* = −10 dB.

Figure 5 and Figure 6 show a comparison of the DoA estimation results for RG signals along the trajectory of the TWAA wearer obtained with (a) the Root-MUSIC module, (b) the MLP-DoA module, and (c) the HSNN-DoA (*ws* = 3) module under increased noise conditions (*SNR* = 0 dB) and significantly increased noise conditions (*SNR* = −10 dB), respectively. These figures show that during the movement of the wearer along the reference trajectory, the values of the angle *θ* estimated with the HSNN-DoA (*ws* = 3) module are significantly closer to the true values than the values estimated with the Root-MUSIC and MLP-DoA modules.

Table 7 shows a comparison of the time (in seconds) required for DoA estimation at 8001 points using Root-MUSIC, MLP-DoA, and HSNN-DoA (*ws* = 3) modules on the reference hardware platform Intel Xeon E-2224 CPU @ 3.4 GHz, 64 GB RAM. The results in Table 7 show that the MLP-DoA module has the highest speed of DoA estimation. The HSNN-DoA module is about eight times slower than the MLP-DoA module because its architecture includes a larger number of neural networks, and the data processing within the simulation software was performed sequentially. The HSNN-DoA module could achieve a speed comparable to the MLP-DoA module if its software implementation allows each neural network to process data in parallel with all other neural networks. Despite the implemented sequential approach to data processing, the HSNN-DoA (*ws* = 3) module is still 10 times faster than the Root-MUSIC module.

Table 8 presents a comparison of the application areas and performance of the HSNN-DoA module (*ws* = 3) with the MLP-DoA module previously developed in [24].

In this study, we analyzed the case of four angular sectors (*N_S_* = 4) and showed that sector division improves robustness under high-noise conditions compared to a single MLP covering the entire angular range. Increasing the number of sectors could further improve accuracy up to a certain limit, but beyond that, performance may degrade due to reduced number of training samples per sector, higher architectural complexity, and increased risk of sector misclassification. Such effects can also increase the “inertia” of the DoA module in tracking rapid changes of the source position, which will be the subject of our future investigations.

The influence of the window length *ws* was analyzed for *ws* = 1, 2, and 3. Results show that increasing *ws* improves robustness against noise, with *ws* = 3 yielding the best performance. However, larger *ws* values also increase the probability of first-level MLPs failing to reach consensus, introducing inertia in the DoA module and slowing its response, which may affect overall accuracy. Further investigation of larger *ws* values will be addressed in future work.

## 5. Conclusions

ANN-based DoA modules are well suited for implementing the smart multi-element TWAA DoA subsystem. Our results show that these DoA modules have significantly higher accuracy in DoA estimation under the textile crumpling conditions caused by the movement of the TWAA wearer than the DoA module implemented with a classical super-resolution algorithm (Root-MUSIC). In addition, ANN-based DoA modules exhibit significantly faster DoA estimation compared to the Root-MUSIC module, making them more suitable for real-time DoA estimation on simple portable platforms. In environments with *SNR* ≥ 5 dB, it is recommended to implement a DoA module with a single MLP network according to the architecture of the proposed MLP-DoA module. In conditions where the noise becomes significant (*SNR* < 5 dB), especially when the noise power exceeds the useful signal power (*SNR* < 0 dB), a sector-based approach is recommended, where the angular space containing the RG is divided into sectors and the DoA module is implemented according to the proposed HSNN-DoA module architecture. In this case, it is recommended that the length of the time integration window is *ws* = 3. Under these conditions, the HSNN-DoA (*ws* = 3) module showed better DoA estimation performance than the Root-MUSIC DoA and MLP-DoA modules.

The results of this study indicate that the proposed HSNN-DoA module can be effectively deployed in wearable healthcare systems, sports and military applications, and mobile IoT platforms, all of which demand compact, lightweight, low-power solutions capable of fast and robust direction finding under high-noise conditions.

Future research will be directed toward determining the optimal values of *N_S_* and *ws*, as well as the implementation of the proposed DoA module on a real portable hardware platform and its integration into the smart TWAA system.

## Figures and Tables

**Table 1:** Testing results for MLP_DOA networks with the best test statistics.

MLP_DoA Network	*WCE* [%]	*ATE* [%]	*r^PPM^*
MLP2-23-23	16.3556	2.1572	0.9942
MLP2-24-20	15.7815	2.1624	0.9942
MLP2-23-19	16.2606	2.1722	0.9941
MLP2-20-20	16.1495	2.1734	0.9941
MLP2-22-18	16.5098	2.1745	0.9941
MLP2-23-20	24.0012	2.1788	0.9941

**Table 2:** Testing results for MLP networks in the sector identification phase with the best accuracy.

Sector 1		Sector 2		Sector 3		Sector 4	
MLP_ID_S_1_	Accuracy [%]	MLP_ID_S_2_	Accuracy [%]	MLP_ID_S_3_	Accuracy [%]	MLP_ID_S_4_	Accuracy [%]
MLP2-18-16	97.8917	MLP2-22-22	97.8167	MLP2-10-6	97.9083	MLP2-9-5	97.9333
MLP2-23-19	97.8583	MLP2-20-10	97.8083	MLP2-10-9	97.8917	MLP2-20-10	97.9167
MLP2-12-12	97.8500	MLP2-10-6	97.8000	MLP2-15-11	97.8750	MLP2-9-6	97.9083
MLP2-17-9	97.8417	MLP2-10-8	97.7917	MLP2-10-8	97.8667	MLP2-10-10	97.9000
MLP2-12-11	97.8333	MLP2-22-16	97.7833	MLP2-23-23	97.8583	MLP2-23-23	97.8917
MLP2-12-10	97.8250	MLP2-8-7	97.7750	MLP2-12-11	97.8500	MLP2-8-7	97.8833

**Table 3:** Testing results for MLP networks in DoA estimation phase with the best test statistics—Sector 1.

MLP_DoA_ S_1_	*WCE* [%]	*ATE* [%]	*r^PPM^*
MLP2-12-8	15.2013	3.1903	0.8289
MLP2-10-8	15.0101	3.2041	0.8276
MLP2-14-12	14.8733	3.2051	0.8268
MLP2-12-12	15.9205	3.2016	0.8267
MLP2-14-11	16.0923	3.2171	0.8264
MLP2-9-5	15.0390	3.2216	0.8263

**Table 4:** Testing results for MLP networks in DoA estimation phase with the best test statistics—Sector 2.

MLP_DoA_ S_2_	*WCE* [%]	*ATE* [%]	*r^PPM^*
MLP2-10-6	4.9530	0.8463	0.9866
MLP2-9-9	5.0364	0.8493	0.9865
MLP2-10-4	5.0137	0.8511	0.9864
MLP2-10-8	5.2496	0.8515	0.9864
MLP2-9-6	5.1556	0.8520	0.9864
MLP2-8-8	5.3224	0.8540	0.9864

**Table 5:** Testing results for MLP networks in DoA estimation phase with the best test statistics—Sector 3.

MLP_DoA_ S_3_	*WCE* [%]	*ATE* [%]	*r^PPM^*
MLP2-9-7	5.1720	0.8438	0.9868
MLP2-9-6	4.9497	0.8446	0.9868
MLP2-15-11	5.2119	0.8448	0.9868
MLP2-10-6	5.1154	0.8472	0.9868
MLP2-10-10	5.0891	0.8479	0.9868
MLP2-12-8	5.1467	0.8506	0.9868

**Table 6:** Testing results for MLP networks in DoA estimation phase with the best test statistics—Sector 4.

MLP_DoA_ S_4_	*WCE* [%]	*ATE* [%]	*r^PPM^*
MLP2-12-11	15.1849	3.2179	0.8345
MLP2-10-10	16.4342	3.2388	0.8331
MLP2-10-9	15.3916	3.2404	0.8331
MLP2-9-6	15.4552	3.2293	0.8328
MLP2-12-8	16.1001	3.2406	0.8324
MLP2-14-12	15.4833	3.2413	0.8321

**Table 7:** DoA estimation time of the Root-MUSIC, MLP-DOA, and HSNN-DoA (*ws* = 3) modules.

DoA Module	Root-MUSIC	MLP-DoA	HSNN-DoA (*ws* = 3)
Time [s]	1.1352	0.0133	0.1106

**Table 8:** Comparison of the HSNN-DoA module (*ws* = 3) with the MLP-DoA module from [24].

DoA Module	Width of Application Sector	Architecture Type	Time-Window-Based Decision	DoA Estimation Accuracy		
				*SNR* ∈ [5 dB, 20 dB]	*SNR* ∈ [−5 dB, 5 dB]	*SNR* ∈ [−15 dB, −5 dB]
MLP-DoA [24]	[−60°, 60°]	Single MLP network	No	High	Medium-low	Unacceptably low
HSNN-DoA (*ws* = 3)	[−80°, 80°]	Hierarchical with multiple sub-sector MLPs	Yes	High	High	Medium-low

## Data Availability

The data presented in this study are available on request from the corresponding author due to privacy reasons.
